# Supplementary material for: HIVprotI: an integrated web based platform for prediction and design of HIV proteins inhibitors
Source: J Cheminform. 2018 Mar 9;10:12. doi: 10.1186/s13321-018-0266-y (PMC5845081; doi:10.1186/s13321-018-0266-y)
Supplement: Supplementary file 2 — Additional file 2. Source code of HIVProtI web server. [file 13321_2018_266_MOESM2_ESM.zip › HIVprotI_Source-code/hivprotI/weka-3-6-6/doc/weka/classifiers/meta/package-tree.html]

weka.classifiers.meta Class Hierarchy


---


|  |  |  |
| --- | --- | --- |
| **Overview** \| **Package** \| Class \| **Tree** \| **Deprecated** \| **Index** \| **Help** \| **Weka's home** | |  |
| **PREV**   **NEXT** | **FRAMES**    **NO FRAMES**     **All Classes** |  |


---


## Hierarchy For Package weka.classifiers.meta

**Package Hierarchies:**: All Packages

---

## Class Hierarchy

- java.lang.Object
  - weka.classifiers.**Classifier** (implements weka.core.CapabilitiesHandler, java.lang.Cloneable, weka.core.OptionHandler, weka.core.RevisionHandler, java.io.Serializable)
    - weka.classifiers.meta.**ClassificationViaClustering**- weka.classifiers.**MultipleClassifiersCombiner**
        - weka.classifiers.**RandomizableMultipleClassifiersCombiner** (implements weka.core.Randomizable)
          - weka.classifiers.meta.**MultiScheme**- weka.classifiers.meta.**Stacking** (implements weka.core.TechnicalInformationHandler)
              - weka.classifiers.meta.**Grading** (implements weka.core.TechnicalInformationHandler)- weka.classifiers.meta.**StackingC** (implements weka.core.OptionHandler, weka.core.TechnicalInformationHandler)
              - weka.classifiers.meta.**Vote** (implements weka.core.TechnicalInformationHandler)
        - weka.classifiers.**SingleClassifierEnhancer**
          - weka.classifiers.meta.**AttributeSelectedClassifier** (implements weka.core.AdditionalMeasureProducer, weka.core.Drawable, weka.core.OptionHandler, weka.core.WeightedInstancesHandler)- weka.classifiers.meta.**ClassificationViaRegression** (implements weka.core.TechnicalInformationHandler)- weka.classifiers.meta.**FilteredClassifier** (implements weka.core.Drawable)- weka.classifiers.**IteratedSingleClassifierEnhancer**
                  - weka.classifiers.meta.**AdditiveRegression** (implements weka.core.AdditionalMeasureProducer, weka.core.OptionHandler, weka.core.TechnicalInformationHandler, weka.core.WeightedInstancesHandler)- weka.classifiers.**RandomizableIteratedSingleClassifierEnhancer** (implements weka.core.Randomizable)
                      - weka.classifiers.meta.**AdaBoostM1** (implements weka.classifiers.Sourcable, weka.core.TechnicalInformationHandler, weka.core.WeightedInstancesHandler)
                        - weka.classifiers.meta.**MultiBoostAB** (implements weka.core.TechnicalInformationHandler)
                        - weka.classifiers.meta.**Bagging** (implements weka.core.AdditionalMeasureProducer, weka.core.TechnicalInformationHandler, weka.core.WeightedInstancesHandler)- weka.classifiers.meta.**Decorate** (implements weka.core.TechnicalInformationHandler)- weka.classifiers.meta.**END** (implements weka.core.TechnicalInformationHandler)- weka.classifiers.meta.**LogitBoost** (implements weka.classifiers.Sourcable, weka.core.TechnicalInformationHandler, weka.core.WeightedInstancesHandler)- weka.classifiers.meta.**RandomCommittee** (implements weka.core.WeightedInstancesHandler)- weka.classifiers.meta.**RandomSubSpace** (implements weka.core.TechnicalInformationHandler, weka.core.WeightedInstancesHandler)- weka.classifiers.meta.**RotationForest** (implements weka.core.TechnicalInformationHandler, weka.core.WeightedInstancesHandler)
                  - weka.classifiers.meta.**OrdinalClassClassifier** (implements weka.core.OptionHandler, weka.core.TechnicalInformationHandler)- weka.classifiers.**RandomizableSingleClassifierEnhancer** (implements weka.core.Randomizable)
                      - weka.classifiers.meta.**CostSensitiveClassifier** (implements weka.core.Drawable, weka.core.OptionHandler)- weka.classifiers.meta.**CVParameterSelection** (implements weka.core.Drawable, weka.core.Summarizable, weka.core.TechnicalInformationHandler)- weka.classifiers.meta.**Dagging** (implements weka.core.TechnicalInformationHandler)- weka.classifiers.meta.**GridSearch** (implements weka.core.AdditionalMeasureProducer, weka.core.Summarizable)- weka.classifiers.meta.**MetaCost** (implements weka.core.TechnicalInformationHandler)- weka.classifiers.meta.**MultiClassClassifier** (implements weka.core.OptionHandler)- weka.classifiers.meta.**RacedIncrementalLogitBoost** (implements weka.core.TechnicalInformationHandler, weka.classifiers.UpdateableClassifier)- weka.classifiers.meta.**ThresholdSelector** (implements weka.core.Drawable, weka.core.OptionHandler)
                      - weka.classifiers.meta.**RegressionByDiscretization**

---


|  |  |  |
| --- | --- | --- |
| **Overview** \| **Package** \| Class \| **Tree** \| **Deprecated** \| **Index** \| **Help** \| **Weka's home** | |  |
| **PREV**   **NEXT** | **FRAMES**    **NO FRAMES**     **All Classes** |  |


---
